# Molecular breeding of *Saccharomyces cerevisiae* with high RNA content by harnessing essential ribosomal RNA transcription regulator

**DOI:** 10.1186/s13568-017-0330-4

**Published:** 2017-02-02

**Authors:** Yu Sasano, Takahiro Kariya, Shogo Usugi, Minetaka Sugiyama, Satoshi Harashima

**Affiliations:** 1 Department of Biotechnology, Graduate School of Engineering, Osaka University, 2-1 Yamadaoka, Suita-shi, Osaka, 565-0871 Japan; 2 Department of Applied Microbial Technology, Faculty of Biotechnology and Life Science, Sojo University, Ikeda 4-22-1, Kumamoto-shi, Kumamoto, 860-0082 Japan

**Keywords:** *S. cerevisiae*, RNA content, Non-transcribed spacer (NTS), rDNA, Rrn5, Fob1

## Abstract

As yeast is commonly used for RNA production, it is industrially important to breed strains with high RNA contents. The upstream activating factor (UAF) plays an important role in transcription of ribosomal RNA (rRNA), a major constituent of intracellular RNA species. Here, we targeted the essential rRNA transcription regulator Rrn5 of *Saccharomyces cerevisiae*, a component of the UAF complex, and disrupted the genomic *RRN5* gene using a helper plasmid carrying an *RRN5* gene. Then we isolated nine suppressor mutants (Sup mutants) of *RRN5* gene disruption, causing deficiency in rRNA transcription. The Sup mutants had RNA contents of approximately 40% of the wild type level and expansion of rDNA repeats to ca. 400–700 copies. Reintroduction of a functional *RRN5* gene into Sup mutants caused a reduction in the number of rDNA repeats to close to the wild type level but did not change RNA content. However, we found that reintroduction of *RRN5* into the Sup16 mutant (in which the *FOB1* gene encoding the rDNA replication fork barrier site binding protein was disrupted) resulted in a significant increase (17%) in RNA content compared with wild type, although the rDNA repeat copy number was almost identical to the wild type strain. In this case, upregulated transcription of non-transcribed spacers (NTS) occurred, especially in the NTS2 region; this was likely mediated by RNA polymerase II and accounted for the increased RNA content. Thus, we propose a novel breeding strategy for developing high RNA content yeast by harnessing the essential rRNA transcription regulator.

**Electronic supplementary material:**

The online version of this article (doi:10.1186/s13568-017-0330-4) contains supplementary material, which is available to authorized users.

## Introduction

Ribonucleic acids (RNA) and nucleotides such as 5′-IMP and 5′-GMP have numerous useful properties. Many researches have shown the efficacy of RNA intake e.g. promotion of tissue differentiation (Rathbone et al. 1992), and immunostimulatory activity (Barbalat et al. 2011). 5`-IMP and 5`-GMP are produced by breaking down of yeast RNA and subsequent conversion of 5`-AMP to 5`-IMP by AMP deaminase and they are well known for “umami” taste and used as food additives (Kurihara and Kashiwayanagi 2000). A budding yeast *Saccharomyces cerevisiae* is the most common microorganism for producing RNA because of its high RNA content (Warner 1999). Because ribosomal RNA (rRNA) accounts for approximately 80% of total RNA in yeast, elevating intracellular rRNA level is the key to construct a yeast strain with high RNA content.

**Fig. 1:**
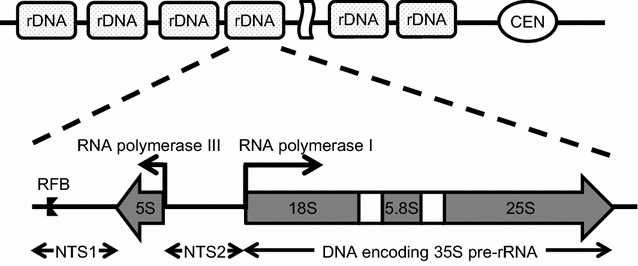
Schematic of a rDNA unit in *S. cerevisiae*. rDNA cluster is located at chromosome XII in *S. cerevisiae*. rDNA repeats consists of 100–150 copies. Each rDNA unit contains two transcribed regions, 35S precursor rRNA and 5S rRNA, and two intergenic non-transcribed spacers, NTS1 and NTS2. RFB, replication fork barrier site

In *S. cerevisiae*, rDNA encoding rRNA are tandemly repeated on chromosome XII and rDNA repeats consists of 100–150 copies (Petes 1979). Each unit is composed of two transcribed regions, 35S precursor rRNA and 5S rRNA, and two intergenic non-transcribed spacers, NTS1 and NTS2 (Fig. 1). The 35S precursor rRNA is normally transcribed by RNA polymerase I (Pol I) and the 5S rRNA is transcribed by RNA polymerase III (Pol III). Transcriptional regulation of 35S rRNA gene is exerted by four factors: core factor (CF), Rrn3, TATA box binding protein (TBP), and upstream activating factor (UAF). CF and Rrn3 are indispensable for basal level transcription of 35S rRNA (Keys et al. 1994; Lalo et al. 1996; Lin et al. 1996; Yamamoto et al. 1996), whereas UAF and TBP are required for high level transcription (Keener et al. 1998; Keys et al. 1996; Steffan et al. 1996). UAF is composed of Rrn5, Rrn9, Rrn10, histone H3, histone H4, and Uaf30 (Goetze et al. 2010). UAF binds to upstream element located at upstream of the 35S pre-rRNA gene to activate the transcription of the 35S precursor RNA gene by recruiting CF and Pol I. In the NTS1 region, there is a replication fork barrier site (RFB), to which fork block protein (Fob1) binds. RFB and Fob1 protein play an important role in maintenance of rDNA repeat copy number at proper level through regulation of rDNA recombination (Kobayashi 2011). It is known that deletion of *FOB1* gene suppresses rDNA recombination and thereby rDNA repeat copy number becomes invariable (Defossez et al. 1999; Johzuka and Horiuchi 2002; Kobayashi et al. 1998).

Fob1 and UAF play an important role in silencing of RNA polymerase II (Pol II)-dependent transcription at 35S rRNA and NTS regions. In UAF-defective mutant caused by disruption of essential component Rrn5 or Rrn9, drastic chromatin remodeling is induced allowing access of Pol II to 35S rRNA promoter, and consequently Pol II-dependent transcription of 35S rRNA, a phenomenon called polymerase switch (PSW) occurs (Goetze et al. 2010; Vu et al. 1999). Furthermore, copy number of rDNA repeat is increased by several fold (Oakes et al. 1999).

Previously, we focused on Rrn10, one of the UAF component and isolated suppressor mutant of *rrn10* disruption. We succeeded in breeding of strains with increased RNA content compared with wild type by reintroduction of functional *RRN10* gene into the suppressor mutants (Chuwattanakul et al. 2011, 2012; Khatun et al. 2013a, b). Because *RRN10* is a non-essential gene, this success prompted us to isolate suppressor mutants of disruption of genes encoding essential UAF components, such as *RRN5*. This was based on the idea that suppressor mutation of disruption of essential genes would have stronger effect than that of non-essential genes in terms of restoration of rDNA transcription activity. Thus, in this study, we isolated suppressor mutants of *rrn5* disruption and by utilizing these mutants we constructed a yeast strain exhibiting high RNA content probably due to increased transcription of NTS region which is normally silenced.

## Materials and methods

### Strains and media

*Saccharomyces cerevisiae* strain SH6471 (NBRP-Yeast, Japan) was used as a parental strain in this study. The detailed information about strains used in this study is shown in Table 1. Yeast cells were grown in YPDA medium consisting of 5% YPD broth (Sigma Aldrich, St. Louis, MO, USA) and 0.04% adenine (Wako, Osaka, Japan) or in SC medium consisting of 0.67% yeast nitrogen base without amino acids (Becton, Dickinson and Company, Franklin Lakes, NJ, USA), 0.2% drop out mix, and 2% glucose. SC medium lacking particular amino acids was used for auxotrophic marker selection. For solid media, 2% agar was used to solidify the medium. *Escherichia coli* DH5α was used for plasmid construction and propagation; *E. coli* recombinant strains were grown in Luria–Bertani (LB) medium containing 100 μg/mL ampicillin.

**Table 1:** *S. cerevisiae* strains and plasmids used in this study

Name	Description
Strain	
SH6471	*MAT*α *ura3*∆*851 his3*∆*200 leu2*∆ *trp1*∆*63 ade2*-*661*
SH8836	*MAT*α *ura3*∆*851 his3*∆*200 leu2*∆ *trp1*∆*63 ade2*-*661* ∆*rrn5::loxP*-*CgTRP1*-*loxP* [pRRN5]
SH8894	*MAT*α *ura3*∆*851 his3*∆*200 leu2*∆ *trp1*∆*63 ade2*-*661* ∆*rrn5::loxP*-*CgTRP1*-*loxP SUP13*
SH8895	*MAT*α *ura3*∆*851 his3*∆*200 leu2*∆ *trp1*∆*63 ade2*-*661* ∆*rrn5::loxP*-*CgTRP1*-*loxP SUP14*
SH8896	*MAT*α *ura3*∆*851 his3*∆*200 leu2*∆ *trp1*∆*63 ade2*-*661* ∆*rrn5::loxP*-*CgTRP1*-*loxP SUP15*
SH8897	*MAT*α *ura3*∆*851 his3*∆*200 leu2*∆ *trp1*∆*63 ade2*-*661* ∆*rrn5::loxP*-*CgTRP1*-*loxP SUP16*
SH8898	*MAT*α *ura3*∆*851 his3*∆*200 leu2*∆ *trp1*∆*63 ade2*-*661* ∆*rrn5::loxP*-*CgTRP1*-*loxP SUP17*
SH8899	*MAT*α *ura3*∆*851 his3*∆*200 leu2*∆ *trp1*∆*63 ade2-661* ∆*rrn5::loxP-CgTRP1-loxP SUP18*
SH8900	*MAT*α *ura3*∆*851 his3*∆*200 leu2*∆ *trp1*∆*63 ade2-661* ∆*rrn5::loxP-CgTRP1-loxP SUP19*
SH8904	*MAT*α *ura3*∆*851 his3*∆*200 leu2*∆ *trp1*∆*63 ade2-661* ∆*rrn5::loxP-CgTRP1-loxP SUP20*
SH8905	*MAT*α *ura3*∆*851 his3*∆*200 leu2*∆ *trp1*∆*63 ade2-661* ∆*rrn5::loxP-CgTRP1-loxP SUP24*
SH30025	Ura^+^ transformants of SH8894 with pRRN5
SH30026	Ura^+^ transformants of SH8895 with pRRN5
SH30027	Ura^+^ transformants of SH8896 with pRRN5
SH30028	Ura^+^ transformants of SH8897 with pRRN5
SH30029	Ura^+^ transformants of SH8898 with pRRN5
SH30030	Ura^+^ transformants of SH8899 with pRRN5
SH30031	Ura^+^ transformants of SH8900 with pRRN5
SH30035	Ura^+^ transformants of SH8904 with pRRN5
SH30036	Ura^+^ transformants of SH8905 with pRRN5
TK1	*MAT*α *ura3*∆*851 his3*∆*200 leu2*∆ *trp1*∆*63 ade2-661* ∆*rrn5::loxP-CgTRP1* ∆*fob1::HIS3* [pRRN5]
TK2	*∆fob1::HIS3* disruptant of SH8894
TK3	*∆fob1::HIS3* disruptant of SH8895
TK4	*∆fob1::HIS3* disruptant of SH8896
TK5	*∆fob1::HIS3* disruptant of SH8897
TK6	*∆fob1::HIS3* disruptant of SH8898
TK7	*∆fob1::HIS3* disruptant of SH8899
TK8	*∆fob1::HIS3* disruptant of SH8900
TK9	*∆fob1::HIS3* disruptant of SH8904
TK10	*∆fob1::HIS3* disruptant of SH8905
TK11	Ura^+^ transformants of TK2 with pRRN5
TK12	Ura^+^ transformants of TK5 with pRRN5
Plasmid	
pRRN5	YCp-*URA3*-*RRN5* (A centromere type plasmid harboring functional *RRN5* gene marked with *URA3* gene)
p2453	pUC18 plasmid harboring *FOB1* gene disruption cassette (a kind gift from T. Kobayashi)

### Disruption of the *RRN5* gene

Because the *RRN5* gene is an essential gene, we introduced a plasmid harboring *RRN5* gene region marked with *URA3* (pRRN5) into SH6471 strain before disruption of the *RRN5* gene. The pRRN5 plasmid was constructed as follows. The *RRN5* gene region including promoter, open reading frame, and terminator, was amplified by PCR using oligonucleotide primer pair, CDR-RRN5-F and CDR-RRN5-R, and genomic DNA of SH6471 strain as a template. The amplified fragment was ligated into the *Sma*I site of pRS316 plasmid (Sikorski and Hieter 1989). The resulting plasmid was named pRRN5. After introduction of pRRN5 into SH6471 strain, the *RRN5* gene was disrupted as follows. The disruption cassette containing *Candida glabrata TRP1* marker gene (*CgTRP1*) was prepared by PCR using oligonucleotide primer pair DR-RRN5-F and DR-RRN5-R, and p3010 plasmid (Sugiyama et al. 2005) as a template. The amplified fragment was introduced into SH6471 strain harboring pRRN5. The obtained transformants (Trp^+^) was checked for disruption of the genomic *RRN5* gene by colony direct PCR using oligonucleotide primer pairs, DR-RRN5-F and DR-RRN5-R, and, CDR-RRN5-F and CDR-RRN5-R (data not shown). The obtained *rrn5* disruptant was named SH8836. Sequence of the oligonucleotide primers used in this study is shown in Additional file 1: Table S1.

### Screening of suppressor mutants

SH8836 strain was cultivated on SC solid medium lacking uracil and tryptophan, and then cells were replicated onto SC solid medium lacking tryptophan and containing 5-fuloroorotic acid (5-FOA) and uracil for curing pRRN5 plasmid. Twenty-four suppressor mutants were isolated and then, absence of *RRN5* gene was confirmed by PCR using oligonucleotide primer pair ORF-RRN5-F and ORF-RRN5-R (data not shown). Finally, nine suppressor mutants were obtained (Sup13, Sup14, Sup15, Sup16, Sup17, Sup18, Sup19, Sup23, and Sup24).

### Measurement of total RNA content

Total intracellular RNA content in yeast was measured by the perchloric acid (PCA) method (Chuwattanakul et al. 2011) with some modifications. Yeast strains were cultivated at 30 °C in SC medium supplemented with appropriate nutrients. Cultured cells were inoculated in 5 mL of the same medium at initial OD_660_ = 0.2 and incubated until OD_660_ reached 1.0. The cell culture was divided into three tubes (1 mL each) and OD_600_ in each tube was measured. After centrifugation of the cell culture (12,000 rpm, 30 s), the supernatant was removed and 1 mL of 0.5 N perchloric acid (Wako, Osaka, Japan) was added and resuspended. After incubation at 70 °C for 20 min, cells were separated by centrifugation (12,000 rpm, 2 min) and absorbance at 260 nm (A_260_) of the supernatant was measured. We calculated total RNA content by using the following equation.

$$ {\text{RNA content }}\left( {{\text{mg}}/{\text{g-Dry cell weight}}} \right)\, = \,\frac{{A_{260} \times 0.0368}}{{OD_{600} \times 0.257 \times 0.001}} $$

Dry cell weight was determined from the standard curve of OD_600_ vs. dry cell weight using SH6471 strain. The mean value and standard deviation were calculated from three divided aliquots of the cell culture.

### Pulsed field gel electrophoresis (PFGE) and Southern hybridization

Chromosomal DNAs from *S. cerevisiae* cultured in SC medium supplemented with appropriate nutrients were embedded in agarose plugs as described by Sheehan and Weiss (Sheehan and Weiss 1990). Chromosomes were separated by CHEF-DR^®^ III pulsed field gel electrophoresis system (Bio-Rad, Hercules, Ca, USA) on 0.8% gel in 1× TAE (0.04 M Tris base, 0.03 M acetic acid, and 1 mM EDTA) buffer at 14 °C. The electrophoretic condition is as follows: voltage, 3 V/cm; switch time, 500 s; angle, 106°; total running time, 48 h. After staining with ethidium bromide, DNA was transferred onto Hybond™-N^+^ membrane (GE Healthcare, Chicago, IL, USA) by capillary blotting. Probe labelling, hybridization, and signal detection were carried out by ECL Direct™ nucleic acid labeling and detection system (GE Healthcare, Chicago, IL, USA). The probe used for detection of rDNA repeat was prepared by PCR amplifying a part of the 5S rRNA gene using oligonucleotide primers SB-probe_5S-F and SB-probe_5S-R, and genomic DNA of SH6471 as a template.

### Quantitative real-time PCR

For determination of rDNA repeat copy number, genomic DNA was extracted by Dr. GenTLE^®^ (from yeast) High Recovery Kit (Takara Bio Inc., Shiga, Japan). Oligonucleotide primers RT-PCR_18S-F and RT-PCR_18S-R were used for amplification of 18S rRNA gene and RT-PCR_ACT1-F and RT-PCR_ACT1-R were for that of *ACT1* gene. For RNA isolation, yeast cells cultivated on SC medium supplemented with appropriate nutrients were collected when OD_600_ reached 1.0. Then, RNA was extracted by RNeasy mini kit (Qiagen, Hilden, Germany). Reverse transcription was performed using QuantiTect Reverse Transcription Kit (Qiagen, Hilden, Germany). Quantitative real-time PCR was performed using SYBR^®^ Premix Ex Taq™ II (Tli RNaseH Plus) (Takara Bio Inc., Shiga, Japan) and Thermal Cycler Dice^®^ Real Time System (Takara Bio Inc., Shiga, Japan). Oligonucleotide primers used for quantitative real-time PCR are listed in Additional file 1: Table S1. Transcription level of *ACT1* gene was used as an internal control.

### Disruption of the *FOB1* gene

The disruption of the *FOB1* gene was performed using p2453 plasmid (Kobayashi and Horiuchi 1996). The 3.4 kb *Eco*RI fragment of p2453 was introduced into SH8836 strain or Sup mutants. Transformants (His^+^) were further subjected to colony direct PCR to check whether the *FOB1* gene was disrupted using an oligonucleotide primer pair FOB1_deletion check-F and FOB1_deletion check-R.

## Results

### Screening of suppressor mutants of *rrn5* disruption

In our previous research, we focused on *RRN10* gene, which is one of the components of UAF, and isolated suppressor mutants of growth defect of *rrn10* disruption. By reintroduction of functional *RRN10* gene into the suppressor mutants, we succeeded in constructing a mutant exhibiting higher RNA content than wild type (Chuwattanakul et al. 2011). This success gave us the idea that isolation and utilization of suppressor mutants of gene disruptant encoding essential UAF components such as Rrn5 would lead to construction of mutants with much higher RNA content. Therefore, we tried to isolate suppressor mutants of *rrn5* disruption. Because *RRN5* gene is an essential gene, a helper plasmid pRRN5 harboring wild type *RRN5* gene expression cassette marked with *URA3* gene was introduced into SH6471 strain prior to *RRN5* gene disruption. Then, genomic *RRN5* gene was disrupted and the obtained disruptant was cultivated on 5-FOA medium in order to remove pRRN5 plasmid. Strains that can grow on 5-FOA medium were expected to have suppressor mutations of *rrn5* disruption. From this screening, we isolated 24 suppressor mutants that could grow on 5-FOA medium. After confirmation of the absence of *RRN5* gene by colony direct PCR, we finally obtained 9 suppressor mutants (Sup mutants) of *rrn5* gene disruption. They were named strains Sup13, Sup14, Sup15, Sup16, Sup17, Sup18, Sup19, Sup21 and Sup24, respectively.

### Characterization of Sup mutants

**Fig. 2:**
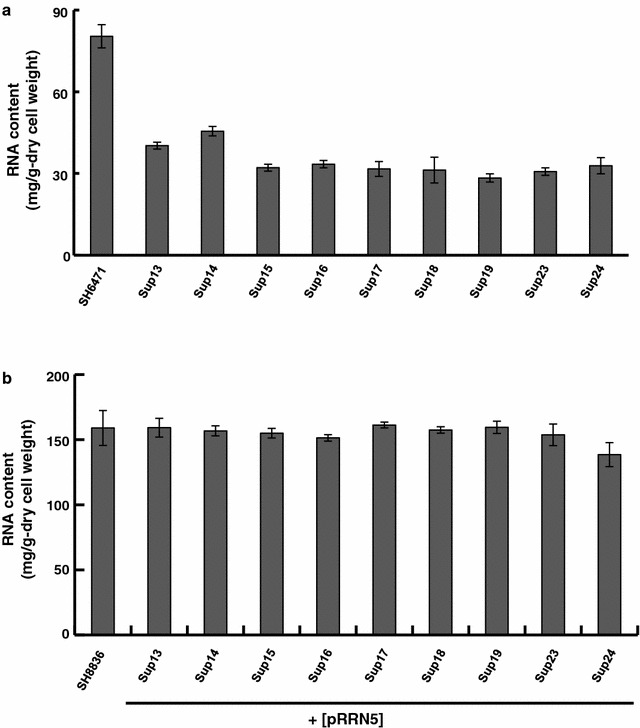
Intracellular total RNA content of Sup mutants. **a** Intracellular total RNA content of nine Sup mutants normalized by dry cell weight. Cells were cultivated in YPDA liquid medium and harvested at middle exponential phase. Intracellular RNA content was measured by PCA method. **b** Intracellular total RNA content of nine Sup mutants harboring pRRN5 plasmid. Cells were cultivated in YPDA liquid medium and harvested at middle exponential phase. Intracellular RNA content was measured by PCA method

We performed dominant/recessive test by crossing Sup mutants and ∆*rrn5* strain with a helper plasmid. This demonstrated that Sup13, Sup14, Sup16, and Sup24 have dominant mutation(s) and Sup15, Sup17, Sup18, Sup19, and Sup23 have recessive mutation(s). (data not shown). In addition, tetrad analysis of the diploids constructed by crossing Sup mutants and ∆*rrn5* strain with a helper plasmid revealed that only Sup15 and Sup16 have a single suppressor gene, whereas the other suppressor mutants have multiple suppressor genes (data not shown). All suppressor mutants could grow on YPDA liquid medium without helper plasmid, although the growth were severely retarded compared with SH8836 strain (Additional file 1: Figure S1). Relative growth rate of suppressor mutants was approximately 10% of the control strain (Additional file 1: Table S2). Total RNA content of the suppressor mutants at middle exponential phase was approximately 40% of the control strain (Fig. 2a). We introduced pRRN5 plasmid into suppressor mutants expecting that transformants will show increased RNA content compared with wild type strain as in the case of suppressor mutants of *rrn10* disruptant (Chuwattanakul et al. 2011). Unexpectedly, total RNA content of all the suppressor mutants having pRRN5 plasmid was almost the same as SH8836 strain (Fig. 2b). This result suggested that the suppression mechanism of *rrn5* disruption was dependent on UAF-deficiency and different from that of *rrn10* disruption.

**Fig. 3:**
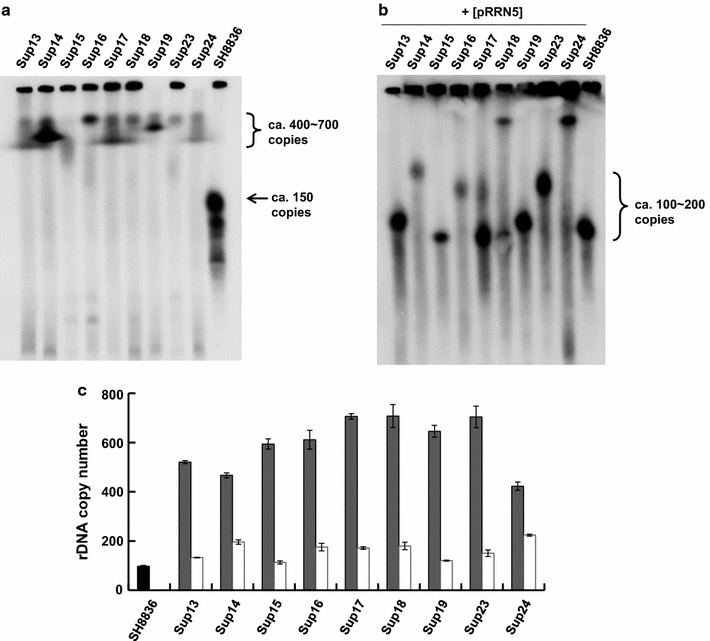
Determination of rDNA copy number of Sup mutants. **a** Sup mutants were subjected to Southern blotting using 5S rRNA gene as a probe after pulsed field gel electrophoresis. The* curly bracket* indicates expanded rDNA repeats in Sup mutants. Broad bands indicate that cell cultures are heterogeneous state. The* arrow* indicates rDNA repeat in SH8836 control strain. **b** Sup mutants harboring pRRN5 were subjected to Southern blotting using 5S rRNA gene as a probe after pulsed field gel electrophoresis. **c** Precise determination of rDNA copy number in Sup mutants by quantitative real-time PCR. A primer pair RT-PCR_18S-F and RT-PCR_18S-R were used for detection of rDNA unit. *ACT1* gene was used as the standard for calculation of rDNA copy number. *Grey bars* Sup mutants, *white bars* Sup mutants harboring pRRN5. The sequence of oligonucleotide primers used in this experiment is listed in Additional file 1: Table S1

Previously, Oakes et al. reported that in UAF-deficient mutant, a switching of 35S rRNA transcription from by RNA polymerase I to by RNA polymerase II (called polymerase switch) and concomitant rDNA repeat expansion occur (Oakes et al. 1999; Vu et al. 1999). Polymerase switch and rDNA expansion is reversible; reintroduction of functional UAF lead to turning back to Pol I-dependent 35S rRNA transcription and rDNA repeat contraction (Oakes et al. 1999; Vu et al. 1999). In order to measure the copy number of rDNA repeat in Sup mutants, we performed pulsed field gel electrophoresis and subsequent Southern blotting using 5S rRNA gene as a probe (Fig. 3a). As with the previous report, Sup mutant showed retarded migration of chromosome XII compared with SH8836 strain, suggesting that expansion of rDNA cluster had occurred. To determine the precise rDNA repeat copy number, quantitative real-time PCR was performed by comparison with *ACT1* gene as a representative of one copy gene (Fig. 3c). The copy number of rDNA repeats in SH8836 strain was approximately 100, while that in Sup mutants varied between 400 and 700. From these results, we concluded that rDNA expansion had occurred in all Sup mutants, and it was suggested that polymerase switch also had occurred in the Sup mutants. In the next step, we measured the copy number of rDNA repeats in Sup mutants harboring pRRN5 plasmid. To this end, we performed PFGE and Southern blotting using 5S rRNA gene as a probe (Fig. 3b) and quantitative real-time PCR (Fig. 3c). The results indicated that rDNA repeat was contracted to almost the same level as wild type (ca. 150-200) and thus, it was suggested that polymerase switching was also abolished by the reintroduction of *RRN5* gene. We speculated that the contraction of rDNA repeats might be the reason for not increasing of total RNA level in Sup mutants harboring pRRN5.

### Combination of suppressor mutation and *fob1* deletion causes concerted increase of intracellular RNA content

**Fig. 4:**
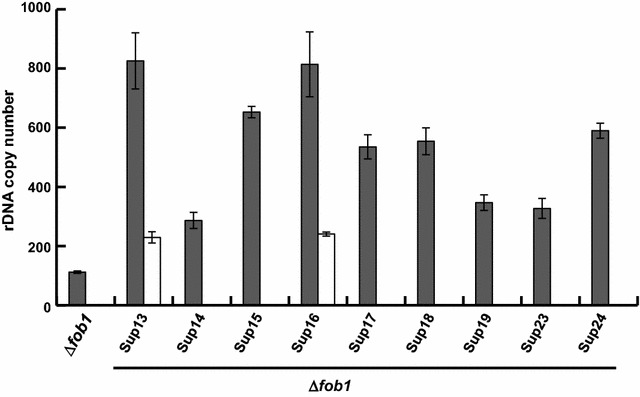
Determination of rDNA copy number of ∆*fob1*Sup mutants. rDNA copy number in ∆*fob1*Sup mutants with or without pRRN5 determined by quantitative real-time PCR. Experiments were performed in triplicates. *Grey bars* ∆*fob1*Sup mutants, *white bars* ∆*fob1*Sup mutants harboring pRRN5

In *S. cerevisiae*, rDNA replication fork barrier site binding protein Fob1 plays an important role in stabilizing rDNA repeat copy number at a defined number (Brewer and Fangman 1988; Kobayashi and Horiuchi 1996; Linskens and Huberman 1988). It was reported that deletion of *FOB1* gene maintains rDNA repeat copy number at a constant level. For this reason, we inferred that if *FOB1* gene is deleted, it might lead to increased total RNA content by the maintenance of expanded rDNA repeats observed in Sup mutant even after introduction of pRRN5. To validate this idea, we disrupted the *FOB1* gene in all Sup mutants. We checked the rDNA repeat copy number in *fob1*-deleted Sup mutants by quantitative real-time PCR (Fig. 4). rDNA repeat copy number varied between 300 copies and 800 copies. Among the mutants, ∆*fob1*Sup13 and ∆*fob1*Sup16 showed the highest copy number (ca. 800 copies).

**Fig. 5:**
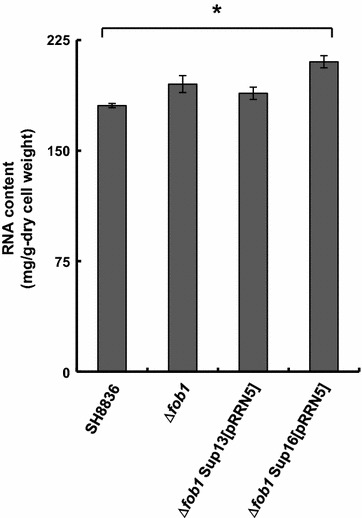
Total RNA content of ∆*fob1*Sup mutants with pRRN5. Intracellular total RNA content of ∆*fob1*Sup13 and ∆*fob1*Sup16 mutants with pRRN5. Cells were cultivated in YPDA liquid medium and harvested at middle exponential phase. Intracellular RNA content was measured by PCA method as described in “Materials and Methods” section. The values are the means and standard deviations of results from three independent experiments. Significant difference of ∆*fob1*Sup16 from SH8836 was confirmed by Student’s t test (P < 0.01)

Then, we introduced pRRN5 plasmid into ∆*fob1*Sup13 and ∆*fob1*Sup16 and measured the rDNA repeat copy number by quantitative real-time PCR (Fig. 4). Unexpectedly, rDNA repeat copy number was decreased to almost the same level as wild type. The reason of this unexpected result is discussed in the Discussion section. However, we happened to discover that total RNA of ∆*fob1*Sup16 harboring pRRN5 (∆*fob1*Sup16[pRRN5]) were significantly increased 16.5% compared with wild type even though rDNA copy number was not increased (Fig. 5), whereas, either ∆*fob1* strain or ∆*fob1*Sup13 harboring pRRN5 (∆*fob1*Sup13[pRRN5]) did not show significant increase of RNA content. We did not measure the RNA content of ∆*fob1*Sup13 and ∆*fob1*Sup16, because growth rate of these mutants was considerably much lower than that of other strains. We took this result to suggest that combination of *fob1* gene disruption and *SUP16* mutation leads to increase of total RNA content even when functional *RRN5* gene is reintroduced. This phenotype is stably maintained because the same results were observed after five times subculture (data not shown).

### Transcription of NTS2 region is remarkably increased in ∆*fob1*Sup16 strain

**Fig. 6:**
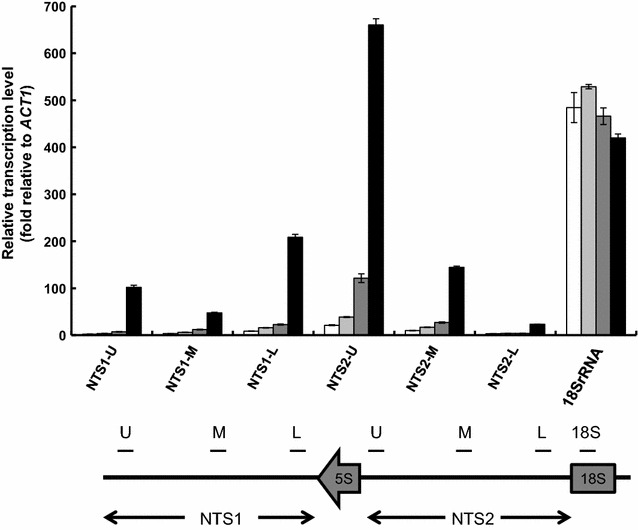
Transcription level of NTS regions. Transcription level of seven sites within rDNA unit was determined by quantitative real-time PCR. Primer pairs used for detection of each site are listed in Additional file 1: Table S1. *White bars*; SH8836. *Light grey bars* Sup16[pRRN5], *dark grey bars* ∆*fob1*; *Black bars* ∆*fob1*Sup16[pRRN5]. The values are the means and standard deviations of results from three independent experiments. The figure below the graph represents the location of each site in this experiment

In order to reveal what kind of RNA species affected the increased intracellular RNA content observed in the ∆*fob1*Sup16[pRRN5] strain, we performed quantitative real-time PCR using primer pairs distinguishing upper, middle, or lower part of the NTS regions and 18S rRNA (Fig. 6). The result indicated that expression levels of 18S rRNA region in all strains tested were almost the same. On the other hand, expression level of NTS1 and NTS2 regions were concertedly increased in ∆*fob1*Sup16[pRRN5] strain, while both ∆*fob1* strain and Sup16[pRRN5] showed only slight increase compared with wild type. In particular, expression of the upper part of NTS2 region (NTS2-U) was markedly increased in the ∆*fob1*Sup16[pRRN5] strain. It was suggested that the transcript observed at NTS2-U is distinct from that observed at 18S rRNA, because almost no expression was observed in the lower part of NTS2 (NTS2-L). We suggest that this increase of NTS2-U is probably attributed to the increased RNA content in the ∆*fob1*Sup16[pRRN5] strain.

## Discussion

Here, we succeeded in construction of a yeast strain with high RNA content by focusing on Rrn5, an essential rRNA transcription regulator. We isolated nine suppressor mutants of *rrn5* disruption. By combination of *FOB1* gene deletion and suppressor mutations, we were able to construct a novel yeast strain showing increased intracellular RNA content.

It was reported that copy number of rDNA repeat is invariable in *fob1* deletion (Defossez et al. 1999; Johzuka and Horiuchi 2002; Kobayashi et al. 1998). However, rDNA copy number was altered to almost the same level as wild type in all ∆*fob1*Sup mutants with pRRN5 plasmid in this study. The reason of this discrepancy might be explained by one or more following reasons. (i) It was reported that when non-coding RNA transcription in NTS1 region driven by Pol II dependent E-PRO promoter is active, cohesins at the NTS region was removed and a recombination called unequal sister-chromatid recombination is induced, resulting in changes in rDNA copy number (Kobayashi 2011; Kobayashi and Ganley 2005). In ∆*fob1*Sup[pRRN5] strain, substantial transcription was observed in NTS1 region as well as NTS2 region (Fig. 6), suggesting that recombination is induced in ∆*fob1*Sup[pRRN5] strain. (ii) Abnormally high copy number of rDNA repeat makes it unstable, thus it is easily popped out to yield extra-chromosomal rDNA circles (ERCs) by Fob1-independent manner. ERC is accumulated only in the mother cell, and its accumulation induces senescence (Kobayashi 2011). Eventually, the number of rDNA copy number becomes closer to that of wild type level.

Within the NTS regions, upstream region of NTS2 (NTS2-U) showed the highest transcription, even higher than 18S rRNA region. This drastically high transcription accounts for the increased intracellular RNA content observed in ∆*fob1*Sup16[pRRN5] strain. In the previous report, Pol II-driven promoter (C-PRO) is located at the NTS2 region (Cesarini et al. 2010; Li et al. 2006). However, it seems unlikely that C-PRO is responsible for increased transcript observed at NTS2-U, because the precise location of C-PRO is at lower part of the NTS2 (corresponding to NTS2-L). Instead, Li et al. reported that an unidentified transcription with the direction from NTS2-U to NTS2-L is observed in the absence of Sir2 (Li et al. 2006). The average size of this cryptic transcript is approximately 1.0 kb, which is comparable to 15% of 35S rRNA transcript (6.9 kb). Therefore, this transcript might be responsible for the increased transcription observed at NTS2-U in ∆*fob1*Sup16[pRRN5] strain because Sir2 recruitment is regulated by Fob1 (Huang and Moazed 2003). It is likely that abundance of Sir2 is reduced in the absence of Fob1 as supported by the observation that the transcription at NTS2-U is slightly increased in ∆*fob1* strain. Thus, it is reasonable that the transcription at NTS2-U is highly induced in ∆*fob1*Sup16[pRRN5] strain by the combination of *fob1* deletion, Sup16 mutation, and *RRN5* reintroduction.

In this study, we did not identify *SUP16* suppressor gene yet. *SUP16* suppressor gene was shown to be dominant from dominant/recessive test by crossing Sup16 strain and ∆*rrn5* strain with a helper plasmid (data not shown) and Sup16[pRRN5] strain showed slight NTS transcription like ∆*fob1* strain. From these results, *SUP16* mutation might be a mutation concerning NTS silencing like Sir2 or other RENT complex factors (Huang and Moazed 2003). Chromatin remodeling factor is also a candidate for *SUP16* because chromatin remodeling is concomitant with NTS transcription. Although *SUP16* suppressor gene is not identified at present, we suggest that it synergistically induces NTS transcription with *fob1* deletion by unknown mechanism even when Rrn5 is functional, and thereby leading to increased intracellular RNA content. Further studies, especially those including identification of the *SUP16* gene are needed to clarify the mechanism.

Previously, we constructed a *S. cerevisiae* strain accumulating approximately 120 mg/g-Dry cell weight RNA using suppressor mutants obtained from *rrn10* disruptant (Chuwattanakul et al. 2011). In this study, we were successful for constructing a strain accumulating approximately 200 mg/g-Dry cell weight RNA by exploiting Rrn5. To our knowledge, this value is higher than any other RNA accumulating yeasts reported so far, although it may not be possible to precisely compare those productivities because culture conditions and medium are different in each report. We propose a novel breeding strategy exploiting transcription of NTS region for high RNA content yeast. Our mutants isolated in this study will be useful not only for an industrial application but for the elucidation of transcriptional regulation of rDNA.

## Additional file

**Additional file 1.** Addiitonal figures and tables.

## Abbreviations

NTS: non transcribed spacer
UAF: upstream activating factor
CF: core factor
RFB: replication fork barrier
5-FOA: 5-fluoroorotic acid
PCA: perchloric acid
PFGE: pulsed field gel electrophoresis
