# Supplementary material for: Molecular breeding of Saccharomyces cerevisiae with high RNA content by harnessing essential ribosomal RNA transcription regulator
Source: AMB Express. 2017 Feb 2;7:32. doi: 10.1186/s13568-017-0330-4 (PMC5289932; doi:10.1186/s13568-017-0330-4)
Supplement: Supplementary file 1 — Additional file 1. Addiitonal figures and tables. [file 13568_2017_330_MOESM1_ESM.pdf]

AMB Express

Molecular breeding of *Saccharomyces cerevisiae* with high RNA content by harnessing essential ribosomal RNA transcription regulator

Yu Sasano[1], Takahiro Kariya[1], Shogo Usugi[1], Minetaka Sugiyama[1] and Satoshi Harashima[2,*]

[1] Department of Biotechnology, Graduate School of Engineering, Osaka University, 2-1 Yamadaoka, Suita-shi, Osaka 565-0871, Japan

[2] Department of Applied Microbial Technology, Faculty of Biotechnology and Life Science, Sojo University, Ikeda 4-22-1, Kumamoto-shi, Kumamoto 860-0082, Japan

* Corresponding author. Tel.: +81 96 326 3837, Fax: +81 96 326 3000

E-mail address: harashima@bio.sojo-u.ac.jp

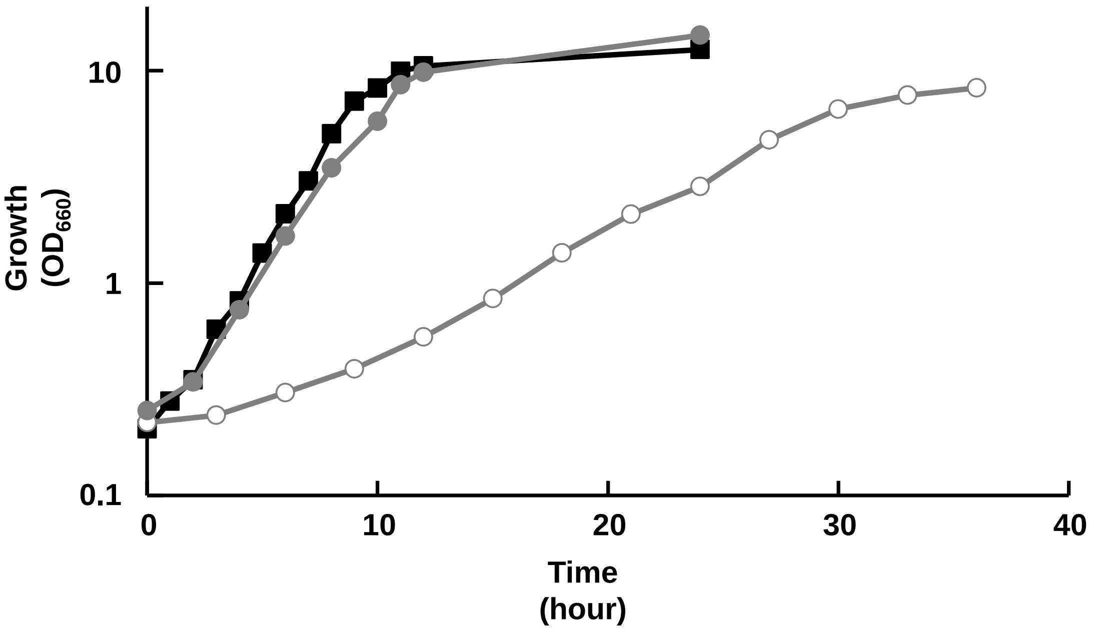

Supplementary Fig. S1 Growth curve of Sup16 and Sup16[pRRN5] strain. SH8836, SH8897 (Sup16), and SH30028 (Sup16[pRRN5]) strains were grown on YPDA liquid medium until stationary phase, then cells were inoculated in YPDA liquid medium at the initial OD_{660} was 0.2. Growth was monitored by measurement of OD_{660}. Similar results were observed in other Sup mutants (data not shown). Black filled square, SH8836; grey filled circle, Sup16[pRRN5]; grey open circle, Sup16.

Supplementary Table S1 Oligonucleotide primers used in this study

Name	Sequence (5`-3`)
CDR-RRN5-F	AGCAAGGGCATTGTATTCCAG
CDR-RRN5-R	GACAAAAGAGAAGAGCTGCT
DR-RRN5-F	GAAGAGCTAGGAAAGCACAGCGCATACGACGGTCACCTAGGCTTCGTACGCTGCAG
DR-RRN5-R	ATGAAAACAAAAATTATGTCCCTGCGCGATTTTTCGCATTGTGTCGCCACTAGTGGATCT
ORF-RRN5-F	ATGGAGCACCAACAATTGCG
ORF-RRN5-R	GCTGATCGGAACAAGTCCGC
SB-probe_5S-F	GCGGCCATATCTACCAGAAA
SB-probe_5S-R	TGCGGAGTTGTAAGATGTAC
RT-PCR_ACT1-F	CGCTCCTCGTGCTGTCTTC
RT-PCR_ACT1-R	TTGACCCATACCGACCATGATA
RT-PCR_18S-F	CCTGAGAAACGGCTACCA
RT-PCR_18S-R	ATTGTCACTACCTCCCTGAATTAAGGA
FOB1_deletion check-F	GGTCTGGGTGTGCAGCTTTC
FOB1_deletion check-R	CACGCCGGTGGACTCTATTC
RT-PCR_NTS1(U)-F	CCGAGCGTGAAAGGATTTGC
RT-PCR_NTS1(U)-R	CACTCATGTTTGCCGCTCTG
RT-PCR_NTS1(M)-F	ATGGCAAGTTCCAGAGAGGCAG
RT-PCR_NTS1(M)-R	GCACTGGCTATTCATCTTGCAC
RT-PCR_NTS1(L)-F	GCGGGAAGGAATAAGAAGCAAC
RT-PCR_NTS1(L)-R	CCTCCATTTCCCTCTCTTCTACG
RT-PCR_NTS2(U)-F	AAGTTGGTCGGTAGGTGGCATG
RT-PCR_NTS2(U)-R	TGCCTGCCACCATCCATTTG

RT-PCR_NTS2(M)-F	CGTTATTGGTAGGAGTGTGGTGG
RT-PCR_NTS2(M)-R	AGACCCTAAAGGGAAATCCATGC
RT-PCR_NTS2(L)-F	TTAAGGCAGAGCGACAGAGAGG
RT-PCR_NTS2(L)-R	TCCTCACACTTGTACTCCATGAC

Supplementary Table S2 Relative growth rate of Sup mutants with or without pRRN5 plasmid

pRRN5 [a]	Strain									
	**SH6471**	**Sup13**	**Sup14**	**Sup15**	**Sup16**	**Sup17**	**Sup18**	**Sup19**	**Sup23**	**Sup24**
-	1.032	0.129	0.122	0.109	0.095	0.081	0.092	0.088	0.117	0.091
+	NA	1.20	1.11	1.29	1.11	0.98	1.00	1.10	1.20	1.18

[a] - represents pRRN5 was not introduced. + represents pRRN5 was introduced.
